# Peripheral blood CD45RO^+^T cells is a predictor of the effectiveness of neoadjuvant chemoradiotherapy in locally advanced rectal cancer

**DOI:** 10.1097/MD.0000000000026214

**Published:** 2021-06-25

**Authors:** Zhiwei Zhai, Zhenjun Wang, Mulan Jin, Kunning Zhang

**Affiliations:** a Department of General Surgery; b Department of Pathology, Beijing Chao-Yang Hospital, Capital Medical University, Beijing, China.

**Keywords:** CD45RO^+^ratio, neoadjuvant therapy, prognosis, rectal cancer

## Abstract

To investigate the relationship between the changes in circulating CD45RO^+^T lymphocyte subsets following neoadjuvant therapy for rectal cancer in patients with locally advanced rectal cancer.

The clinicopathological data of 185 patients with rectal cancer who received neoadjuvant therapy in the General Surgery Department of Beijing Chaoyang Hospital affiliated to Capital Medical University from June 2015 to June 2017 were analyzed. Venous blood samples were collected 1 week before neoadjuvant therapy and 1 week before surgery, and the expression of CD45RO^+^T was detected by flow cytometry. The receiver operating characteristic curve analysis was used to determine the optimal cut-off point of CD45RO^+^ratio. Log-rank test and multivariate Cox regression were used to analyze the overall survival rate (OS) and disease-free survival rate (DFS) associated with CD45RO^+^ratio.

Circulating CD45RO^+^ratio of 1.07 was determined as the optimal cut-off point and CD45RO^+^ratio-high was associated with lower tumor regression grade grading (*P* = .031), T stage (*P* = .001), and tumor node metastasis (TNM) stage (*P* = .012). The 3-year DFS and OS rate in the CD45RO^+^ratio-high group was significantly higher than that in the CD45RO^+^ratio-low group (89.2% vs 60.1%, *P*<.001; 94.4% vs 73.2%, *P*<.001). The multivariate Cox analysis revealed that elevated CD45RO^+^ratio was an independent factor for better DFS (OR, 0.339; 95% CI, 0.153–0.752; *P* = .008) and OS (OR, 0.244; 95% CI,0.082–0.726; *P* = .011).

Circulating CD45RO^+^ratio could predict the tumor regression grade of neoadjuvant therapy for rectal cancer, as well as long-term prognosis. These findings could be used to stratify patients and develop alternative strategies for adjuvant therapy.

## 1 Introduction

At present, colorectal cancer is still the third-largest malignancy with reference to the incidence and mortality worldwide.^[1]^ Preoperative neoadjuvant chemoradiotherapy is currently recommended as the standard treatment for locally advanced rectal cancer, as it can effectively control the local recurrence rate.^[2,3]^

It is generally believed that radiotherapy and chemotherapy can inhibit the immune response of patients. However, previous studies have found that radiotherapy and chemotherapy could promote immune response and cause tumor regression.^[4,5]^ Some studies reported that circulating lymphocytes of patients before neoadjuvant therapy were correlated with the efficacy of neoadjuvant therapy. The lower total number of lymphocytes was associated with the lower ratio of lymphocytes to neutrophils or monocytes, which in turn was associated with patients being less sensitive to neoadjuvant therapy and with the worse prognosis.^[6–10]^ Yet, these are studies on circulating baseline levels of patients before neoadjuvant therapy, and there are few studies on the dynamic changes of lymphocytes. At the same time, studies on lymphocyte subtypes are also very rare.

Memory T lymphocytes with CD45RO^+^ phenotype^[11]^ can secrete interferon (IFN)-γ, CC-type chemokine 4 (CCL4), C-type chemokine 1 (XCL1), and other cytokines to exert a direct or indirect antitumor effect.^[12]^ Our previous studies have found that the density of locally infiltrating CD45RO^+^T lymphocytes was associated with the sensitivity of neoadjuvant therapy and the prognosis of patients with rectal cancer.^[13]^ However, the relationship between circulating CD45RO^+^T lymphocytes and neoadjuvant therapy has not been reported. Therefore, in this study, the level of circulating CD45RO^+^T lymphocytes before and after neoadjuvant therapy was detected to investigate the relationship between the changes of CD45RO^+^T lymphocytes and the efficacy of neoadjuvant therapy for rectal cancer.

## 2 Materials and methods

### 2.1 Patients

A total of 185 patients with rectal cancer admitted to the General Surgery Department of Beijing Chaoyang Hospital affiliated to Capital Medical University from June 2015 to June 2017 were selected. Inclusion criteria were the following: rectal adenocarcinoma confirmed by pathology, which was within 12 cm from the anal verge; patients diagnosed with cT_3∼4_N_0_ or cT_1∼__4_N_1-2_ by magnetic resonance imaging or transrectal ultrasound; distant metastasis was excluded by the chest and abdominal computed tomography examination; single rectal tumor confirmed by colonoscopy; patients who did not receive preoperative chemotherapy, pelvic radiotherapy, or immunotherapy; no previous history of other tumors; patients who scored 0 to 1 point by the Eastern Cooperative Oncology Group;^[14]^ patients who did not have any serious cardiac, pulmonary, renal, or other complications; those who were able to tolerate chemoradiotherapy. Exclusion criteria were: patients who underwent radical resection of rectal cancer before and had local recurrence this time; patients who could not complete the course of neoadjuvant therapy; patients with incomplete clinicopathological data.

Informed consent was obtained from all participants before treatment. The study was approved by the ethics committee of Beijing Chao-Yang Hospital, Capital Medical University.

### 2.2 Treatment

All patients were treated with three-dimensional intensity-modulated radiation therapy. The target areas of radiotherapy, including primary tumor area and metastatic lymph node area, were defined by experienced radiologists. The dose of radiotherapy was 50.4 Gy/28f. Oral capecitabine chemotherapy was conducted during radiotherapy at the dose of 825 mg/m^2^, orally, twice a day. Surgery was performed after 6 to 8 weeks rest after radiotherapy. The surgery followed the principle of total mesorectal excision, and abdominoperineal resection, anterior rectal resection, or Hartmann surgery were performed depending on the location of the tumor. Postoperative adjuvant chemotherapy of the CapeOX regimen was given to complete the adjuvant therapy in the perioperative period for 6 months. Postoperative adjuvant chemotherapy regimen: oxaliplatin 130 mg/m^2^ on day 1, capecitabine 1000 mg/m^2^, twice a day on days 1 to 14, every 3 weeks.

### 2.3 Pathological assessment

The tumor stage was evaluated by 2 pathologists according to the 7th edition of the American Joint Committee on Cancer tumor node metastasis (TNM) system. The tumor regression grade (TRG) was used to grade tumor response as recommended by the American Joint Committee on Cancer Staging Manual modified from Ryan R.^[15]^ No remaining viable cancer cells were defined as complete response (TRG 0). Only small clusters or single cancer cells remaining were defined as a moderate response (TRG 1). Residual cancer remaining with predominant fibrosis was defined as a minimal response (TRG 2). Extensive residual cancer was defined as a poor response (TRG 3).

### 2.4 Blood sample and flow cytometry

Peripheral venous blood samples were collected within 7 days before the start of neoadjuvant therapy and 7 days before rectal surgery. Circulating lymphocytes were evaluated by routine clinical flow cytometry. We added the sample into a tube with 2.5 μg of Human Becton Dickinson (BD) Fc Block for 10 minutes. Then, samples were incubated with 10 μL of the fluorochrome-conjugated antibody for 20 minutes at room temperature in phosphate buffer solution (PBS) containing 0.1% (wt/vol) BSA and 0.1% NaN3.

**Figure 1:**
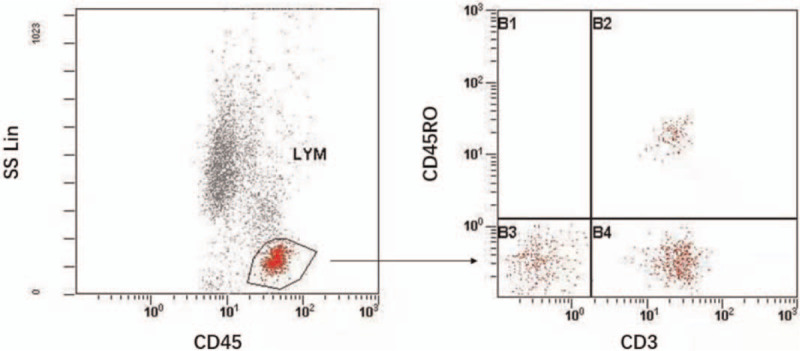
Flow cytometry and gating of CD45RO populations in peripheral blood of the patients.

Phenotypes of the T-cell populations were obtained with PE-labeled anti-CD45, APC-Cy7-labeled anti-CD3 antibody plus combinations of fluorescein isothiocyanate (FITC)-labeled CD45RO. The antibodies were purchased from BD Biosciences (San Jose, CA): anti-CD45 (HI30), anti-CD3 (SK7), anti-CD45RO (UCHL1). We added a Lysis buffer (Biolegend, 420301) into the samples for 10 minutes. Next, cells were centrifuged at 270 g for 5 minutes and resuspended in fluorescence activated cell sorting (FACS) staining buffer. Subsequently, the data were acquired on a BD FACSCanto and analyzed with FlowJo software (Treestar) (Fig. 1).

### 2.5 Follow-up

Patients were followed up by a colorectal surgeon every 3 months for the first 2 postoperative years and every 6 months for the next 3 years. Evaluation at each visit included physical examination, digital rectal examination, complete blood count, blood chemistry analysis, and carcinoembryonic antigen measurement. Chest radiography and computed tomography of the abdomen and pelvis were conducted every 6 months after treatment. Colonoscopy was performed every year after surgery. Overall survival (OS) referred to the time elapsed between the date of surgery to the date of death or last follow-up, while disease-free survival (DFS) referred to the time elapsed between the date of surgery to the date of disease recurrence or last follow-up.

### 2.6 Statistical analyses

All statistical analyses were performed using the IBM SPSS Statistics 20.0 software (SPSS Inc, Chicago, IL). Categorical variables were analyzed with Pearson *χ*^2^ or Fisher exact test. A receiver operating characteristic curve analysis was performed to define the optimal cut-off value of the CD45RO^+^ratio for predicting 3-year OS. Survival rates of different groups were calculated by the Kaplan–Meier method, and the differences in the survival curves were compared with the log-rank test. The Cox proportional hazard regression models were used for univariable and multivariable analyses. A *P* value of <.05 was considered to be statistically significant.

## 3 Results

### 3.1 Patient characteristics

One hundred eighty-five patients were reviewed based on the inclusion and exclusion criteria. The median age was 59 years (range, 28–76 years), and 59.5% (110/185) of patients were male, and 40.5% (75/185) of patients were female. The median distance of the tumor from the anal verge was 5 cm (range, 1–11 cm). The initial clinical prestage was cII in 29.7% (55/185) and cIII in 70.3% (130/185) of cases. Among these patients, 68.1% (126/185) and 27.0% (50/185) of patients underwent low anterior resection and abdominoperineal resection, while the other 9 (4.9%) patients underwent a Hartmann procedure. The distribution of ypTNM stages was as follows: complete pathologic response found in 10.8% patients (20/185); stage I in 26.5% patients (49/185); stage II in 24.9% patients (46/185); stage III in 37.8% patients (70/185). Lymphovascular invasion was observed in 12.4% (23/185) of patients.

### 3.2 Determination of the cut-off point

**Figure 2:**
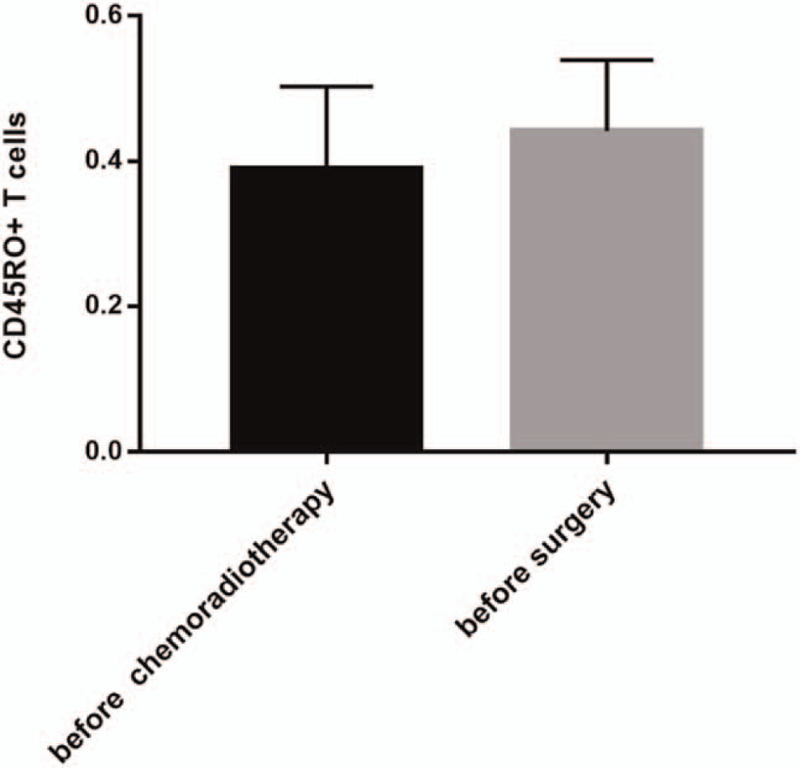
The changes of CD45RO^+^ T cells ratio before the start of chemoradiotherapy and before rectal surgery.

The average values of the CD45RO^+^ T cells in peripheral blood lymphocytes of the patients before surgery were higher than that before neoadjuvant treatment (44.2% vs 38.9%, *P* = .032, Fig. 2).

The ratio of the value after neoadjuvant therapy to that before neoadjuvant therapy was used to reflect the changes of circulating CD45RO^+^T lymphocytes.

**Figure 3:**
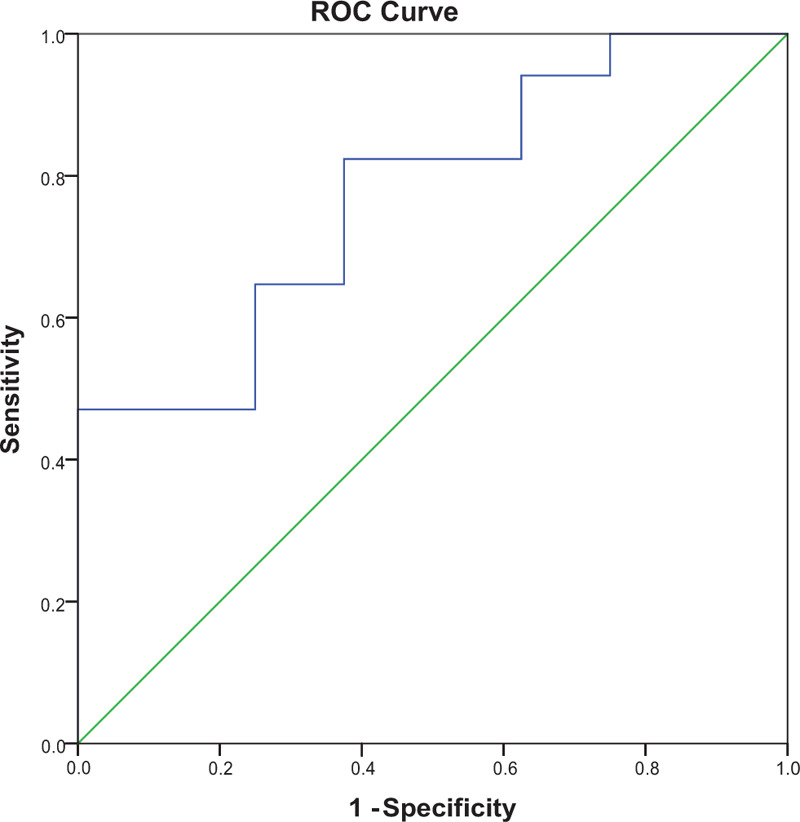
ROC curve for determining the cut-off point of circulating CD45RO^+^ratio before and after neoadjuvant therapy. ROC = receiver operating characteristic.

The optimal cut-off value of the CD45RO^+^ratio was 1.07, corresponding to maximum sensitivity and specificity (0.824 and 0.695, respectively) of the CD45RO^+^ratio for predicting 3-year OS in receiver operating characteristic analysis. The area under the curve was 0.772 for 3-year OS (95% confidence interval [CI], 0.533–0.946, *P* = .031). Patients were then assigned either to the CD45RO^+^ratio-high (CD45RO^+^ratio≥1.07) group or the CD45RO^+^ratio-low (CD45RO^+^ratio<1.07) group (Fig. 3).

### 3.3 Association between CD45RO^+^ratio and clinicopathological characteristics

The association between CD45RO^+^ratio and patient characteristics is shown in Table 1. CD45RO^+^ratio was significantly correlated with lesser-advanced ypT stage (*P* = .001) and ypTNM stage (*P* = .012). The incidence of higher differentiation grade (*P* = .034) and TRG (*P* = .043) was higher in patients with CD45RO^+^ratio-low than in those with CD45RO^+^ratio-high. CD45RO^+^ratio was not significantly correlated with other clinicopathological factors.

**Table 1:** Association between CD45RO^+^ratio and clinicopathological characteristics.

	CD45RO^+^ratio		
	<1.07	≥1.07	
Variables	n=95	n=90	*P* value
Sex			
Male	57 (60.0%)	53 (58.9%)	.878
Female	38 (40.0%)	37 (41.1%)	
Age (yrs)			
<60	65 (68.4%)	60 (66.7%)	.799
≥60	30 (31.6%)	30 (33.3%)	
Distance from anal verge (cm)			
<5	37 (38.9%)	35 (38.9%)	.993
≥5	58 (61.1%)	55 (61.1%)	
pre-CRT CEA level (ng/mL)			
<5	47 (49.5%)	47 (52.2%)	.709
≥5	48 (50.5%)	43 (47.8%)	
Tumour differentiation			
G1-2	58 (61.1%)	68 (75.6%)	**.034**
G3-4	37 (38.9%)	22 (24.4%)	
Clinical stage			
II	29 (30.5%)	26 (28.9%)	.808
III	66 (69.5%)	64 (71.1%)	
Surgery			
Anterior resection	61 (64.2%)	65 (72.2%)	.243
Nonanterior resection	34 (35.8%)	25 (27.8%)	
TRG			
TRG0-1	21 (22.1%)	32 (35.6%)	**.043**
TRG2-3	74 (77.9%)	58 (64.4%)	
ypT			
T0	6 (6.3%)	3 (3.3%)	**.001**
T1–2	21 (22.1%)	42 (46.7%)	
T3–4	68 (71.6%)	45 (50.0%)	
ypN			
N0	50 (52.6%)	60 (66.7%)	.052
N1–2	45 (47.4%)	30 (33.3%)	
ypTNM stage			
ypCR	9 (9.5%)	11 (12.2%)	**.012**
I	16 (16.8%)	33 (36.7%)	
II	27 (28.4%)	19 (21.1%)	
III	43 (45.3%)	27 (30.0%)	
LVI			
Negative	82 (86.3%)	80 (88.9%)	.596
Positive	13 (13.7%)	10 (11.1%)	

CEA= carcinoembryonic antigen, CRT = chemoradiotherapy, LVI = lymphovascular invasion. CD45RO^+^ratio-high was associated with lower tumor regression grade grading (*P* = .031), T stage (*P* = .001), and TNM stage (*P* = .012).

### 3.4 Analysis of the prognostic impact of the CD45RO^+^ratio on DFS and OS

The follow-up time of 185 patients ranged from 6.9 to 59.1 months (median, 38.6), and the 3-year DFS and OS were 75.8% and 84.2%, respectively.

**Figure 4:**
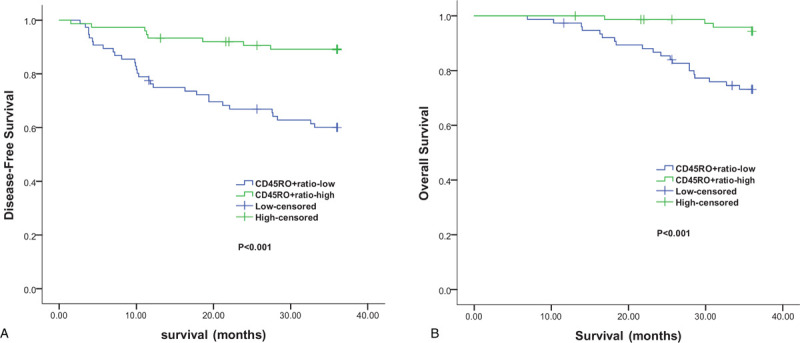
Kaplan–Meier survival curves according to CD45RO^+^ratio level. A, Disease-free survival. B, Overall survival.

Kaplan–Meier analysis indicated that the 3-year DFS and OS rate in the CD45RO^+^ratio-high group was significantly higher than that in the CD45RO^+^ratio-low group (89.2% vs 60.1%, *P*<.001; 94.4% vs 73.2%, *P*<.001) (Fig. 4).

The univariate analysis showed that CD45RO^+^ratio, tumor differentiation, ypT stage, and ypN stage were related to 3-year DFS rate (all *P*<.05) (Table 2). On the other hand, CD45RO^+^ratio, tumor differentiation, TRG, ypT stage, and ypN stage were related to 3-year OS rate (all *P*<.05) (Table 3). The multivariate analysis showed that CD45RO^+^ratio (odds ratio [OR], 0.339; 95% CI, 0.153–0.752; *P* = .008), and ypN stage (OR, 2.242; 95% CI, 1.103–4.566; *P* = .026) were significant prognostic factors for 3-year DFS rate (Table 2). Similarly, CD45RO^+^ratio (OR, 0.244; 95% CI, 0.082–0.726; *P* = .011) and tumor differentiation (OR, 2.787; 95% CI, 1.099–7.064; *P* = .031) were also defined as independent factors for 3-year OS rate (Table 3).

**Table 2:** Univariate and multivariate analysis of factors affecting DFS.

	Univariate analysis		Multivariate analysis	
Variables	Odds ratio (95% CI)	*P* value	Odds ratio (95% CI)	*P* value
Sex	0.794 (0.408–1.544)	.496		
Age	1.023 (0.994–1.054)	.126		
Distance from anal verge (cm)	1.019 (0.974–1.067)	.405		
pre-CRT CEA level (ng/mL)	1.207 (0.638–2.285)	.563		
Tumour differentiation	3.351 (1.778–6.315)	<**.001**	1.847 (0.935–3.648)	.077
Clinical stage	1.608 (0.857–3.017)	.139		
Surgery	0.997 (0.984–1.011)	.703		
TRG	0.979 (0.954–1.005)	.144		
ypT	6.141 (2.181–17.289)	**.001**	2.872 (0.951–8.673)	.061
ypN	3.365 (1.729–6.551)	<**.001**	2.242 (1.103–4.556)	**.026**
LVI	0.669 (0.295–1.517)	.336		
CD45RO^+^ratio	0.228 (0.104–0.497)	<**.001**	0.339 (0.153–0.752)	**.008**

CEA= carcinoembryonic antigen, CRT = chemoradiotherapy, LVI = lymphovascular invasion, TRG = tumor regression grade. The multivariate Cox analysis revealed that elevated CD45RO^+^ratio was an independent factor for better DFS (OR, 0.339; 95% CI, 0.153–0.752; *P* = .008).

**Table 3:** Univariate and multivariate analysis of factors affecting OS.

	Univariate analysis		Multivariate analysis	
Variables	Odds ratio (95% CI)	*P* value	Odds ratio (95% CI)	*P* value
Sex	0.795 (0.343–1.841)	.592		
Age	1.037 (0.998–1.077)	.060		
Distance from anal verge (cm)	1.018 (0.961–1.079)	.536		
pre-CRT CEA level (ng/mL)	1.000 (0.988–1.013)	.963		
Tumor differentiation	2.285 (1.511–3.455)	<**.001**	2.787 (1.099–7.064)	**.031**
Clinical stage	1.547 (0.706–3.390)	.276		
Surgery	0.980 (0.881–1.090)	.708		
TRG	0.967 (0.936–0.999)	**.043**	0.980 (0.948–1.103)	.235
ypT	4.919 (1.472–16.476)	**.010**	1.758 (0.462–6.690)	.408
ypN	2.818 (1.245–6.378)	**.013**	1.739 (0.695–4.351)	.237
LVI	0.723 (0.248–2.108)	.553		
CD45RO^+^ratio	0.178 (0.061–0.522)	**.002**	0.244 (0.082–0.726)	**.011**

CEA= carcinoembryonic antigen, CRT = chemoradiotherapy, LVI = lymphovascular invasion, TRG = tumor regression grade. The multivariate Cox analysis revealed that elevated CD45RO^+^ratio was an independent factor for better OS (OR, 0.244; 95% CI, 0.082–0.726; *P* = .011).

## 4 Discussion

The sensitivity of rectal cancer to neoadjuvant therapy depends on the sensitivity of rectal tumor cells to radiation, as well as on the tumor microenvironment and the immune state of the body.^[4,16]^ A meta-study revealed that in the current published studies on solid tumors, CD45RO^+^ T cell infiltration was significantly associated with improved OS and DFS.^[17]^ Our previous studies also confirmed that the density of locally infiltrating CD45RO^+^T lymphocytes was associated with the sensitivity of neoadjuvant therapy and the prognosis of patients with rectal cancer.^[13]^ As a marker of memory T lymphocytes, CD45RO can be rapidly activated after re-exposure to the same antigen, releasing Th1 cytokines such as IL-1, IL-4, IFN-γ, etc to assist the rapid synthesis and secretion of IgG antibodies by B lymphocytes, thus enhancing humoral and cellular immunity of the body.^[18]^ Still, the results of studies on tumor-infiltrating lymphocytes from postoperative pathological specimens cannot prospectively predict the efficacy of neoadjuvant therapy. Hsu et al^[19]^ found that in glioblastoma patients receiving immunotherapy, the overlap of tumor-infiltrating T lymphocyte receptor sequence and circulating T lymphocyte could predict whether the patient's body would develop an immune response, thus suggesting that circulating lymphocytes are related to tumor-infiltrating lymphocytes. Detection of circulating lymphocytes can reflect the immune status of the body.

Recently, some researchers have explored the relationship between circulating lymphocyte subsets and the prognosis of solid tumors. Yang et al^[20]^ found that circulating CD4^+^ naive/memory ratio could be used as a prognostic indicator for non-small cell lung cancer and personalized treatment strategies could be optimized according to the detection results. In studies on pancreatic cancer, Hang et al^[21]^ found that circulating baseline CD45RO^+^T before treatment was associated with patients’ OS (*P* = .036). At the same time, multivariate analysis showed that CD4^+^naive/memory ratio was an independent prognostic factor for patients (HR 1.427, 95% CI 1.033–1.973, *P* = .031). At present, few studies investigated the relationship between circulating lymphocytes and neoadjuvant therapy in rectal cancer, and all of which focused on the baseline level of circulating lymphocytes before neoadjuvant therapy.^[6–10]^ Therefore, this study dynamically studied the relationship between peripheral lymphocytes and neoadjuvant therapy by detecting the changes of CD45RO^+^T lymphocytes during neoadjuvant therapy. Moreover, peripheral blood detection is simple, low cost, and can be used to achieve standardized quality control, which is convenient for clinical efficacy prediction in patients.

Survival analysis in this study revealed that the 3-year DFS (89.2% vs 60.1%, *P* < .001) and OS (94.4% vs 73.2%, *P* < .001) in the CD45RO^+^ratio-high group was significantly prolonged compared with the control group. The multivariate analysis revealed that CD45RO^+^ratio was an independent prognostic factor for 3-year DFS (OR, 0.339; 95% CI, 0.153–0.752; *P* = .008) and OS (OR, 0.244; 95% CI, 0.082–0.726; *P* = .011). The results of this study are consistent with those of other studies on solid tumors.^[20,21]^ These findings suggest that the proportion of circulating lymphocytes, rather than absolute count, can better reflect the body's systemic immune response to tumors. This may be because the absolute number of T cells in different phenotypes is also affected to varying degrees by other factors such as age,^[11]^ while the proportion of lymphocytes can reduce this effect.

CD45RO^+^ratio in the present study was found to be correlated with TRG and negatively correlated with ypTNM staging. We speculated that the sensitivity of the tumor to neoadjuvant therapy and the host's immune response might be mutually causal. After neoadjuvant therapy, tumors with high sensitivity to treatment were more likely to release more tumor antigens. Correspondingly, high levels of tumor antigen continuously stimulate the host's immune response, leading to tumor regression.

Currently, it is believed that patients with decreased circulating lymphocyte caused by chemoradiotherapy have a poor prognosis, which may be due to the destruction of immune function by radiotherapy and chemotherapy.^[22,23]^ Nevertheless, some studies also found that the decrease rate of circulating lymphocytes was related to the efficacy of neoadjuvant therapy, and the higher the decrease rate was, the more obvious the efficacy was.^[24]^ DeMaria et al^[25]^ argued that radiotherapy could induce antigen released during the death of cancer cells, which in turn combines with pro-inflammatory signals that trigger the innate immune system and activate tumor-specific T cells, converting them into in situ tumor vaccines. For chemotherapy, Zitvogel et al^[26]^ concluded that effective chemotherapy could stimulate immune effector cells and eliminate immunosuppressive cells. Evidence showed that traditional chemotherapy and radiotherapy regimens could induce dendritic cell activation, enhance antigen presentation, selectively remove immunosuppressive cells, and restore the immunosuppressive state caused by the tumor.^[27]^ The strength of the body's antitumor immunity is not only reflected from the increase of the number of immune cells but, more importantly, is from the enhancement of anti-tumor activity. Recently, it has been found that the anti-tumor effect of IFN-γ secreted by infiltrating lymphocytes around microsatellite stable colorectal cancer was relatively weak, and the author believed that the local anti-tumor immunity could not be reflected only from the number of infiltrating lymphocytes.^[28]^ Future studies should study the function of CD45RO^+^T lymphocytes.

The present study has some limitations that need to be pointed out. First, this is a retrospective study with a low level of evidence-based medicine, short follow-up time, and no long-term survival data. Second, only circulating CD3^+^CD45RO^+^T cells were detected. To better determine the role of lymphocytes, future studies should use more detailed lymphocyte classification, including CD4^+^CD45RO^+^T cells and CD8^+^CD45RO^+^T cells, as well as studies on immune function. Third, some other factors, such as inflammation, ischemia, and age, were not considered in this study.

## 5 Conclusion

Circulating CD45RO^+^ratio is associated with TRG of neoadjuvant therapy for rectal cancer, which provides an idea for predicting the sensitivity of neoadjuvant therapy for rectal cancer. Meanwhile, CD45RO^+^ratio, which is an independent prognostic factor for patients with rectal cancer treated with neoadjuvant therapy, can be used by doctors in making individualized treatment plans for patients.

## Author contributions

**Conceptualization:** Kunning Zhang.

**Data curation:** Zhiwei Zhai, Kunning Zhang.

**Formal analysis:** Zhiwei Zhai, Kunning Zhang.

**Investigation:** Zhiwei Zhai.

**Methodology:** Zhiwei Zhai, Mulan Jin, Kunning Zhang.

**Project administration:** Zhenjun Wang, Mulan Jin.

**Software:** Zhiwei Zhai.

**Supervision:** Zhenjun Wang, Mulan Jin, Kunning Zhang.

**Writing – original draft:** Zhiwei Zhai, Kunning Zhang.

**Writing – review & editing:** Zhiwei Zhai, Kunning Zhang.
